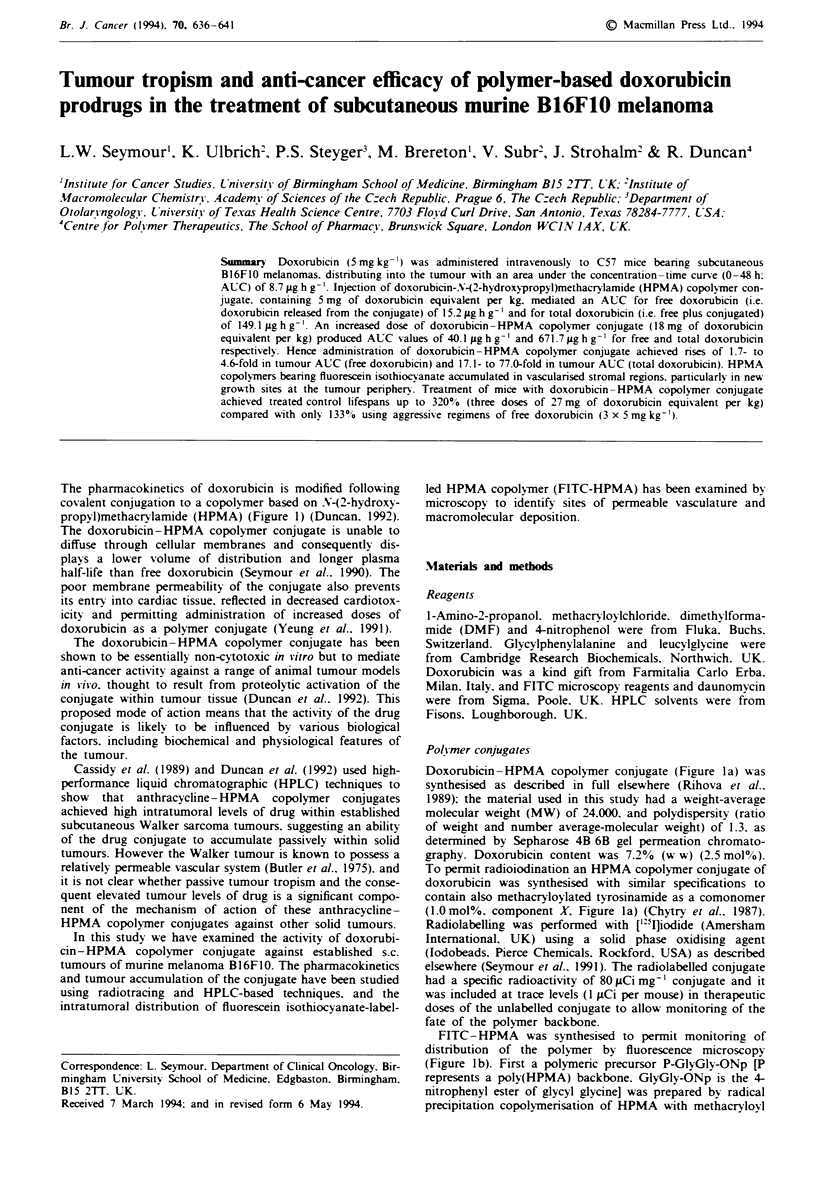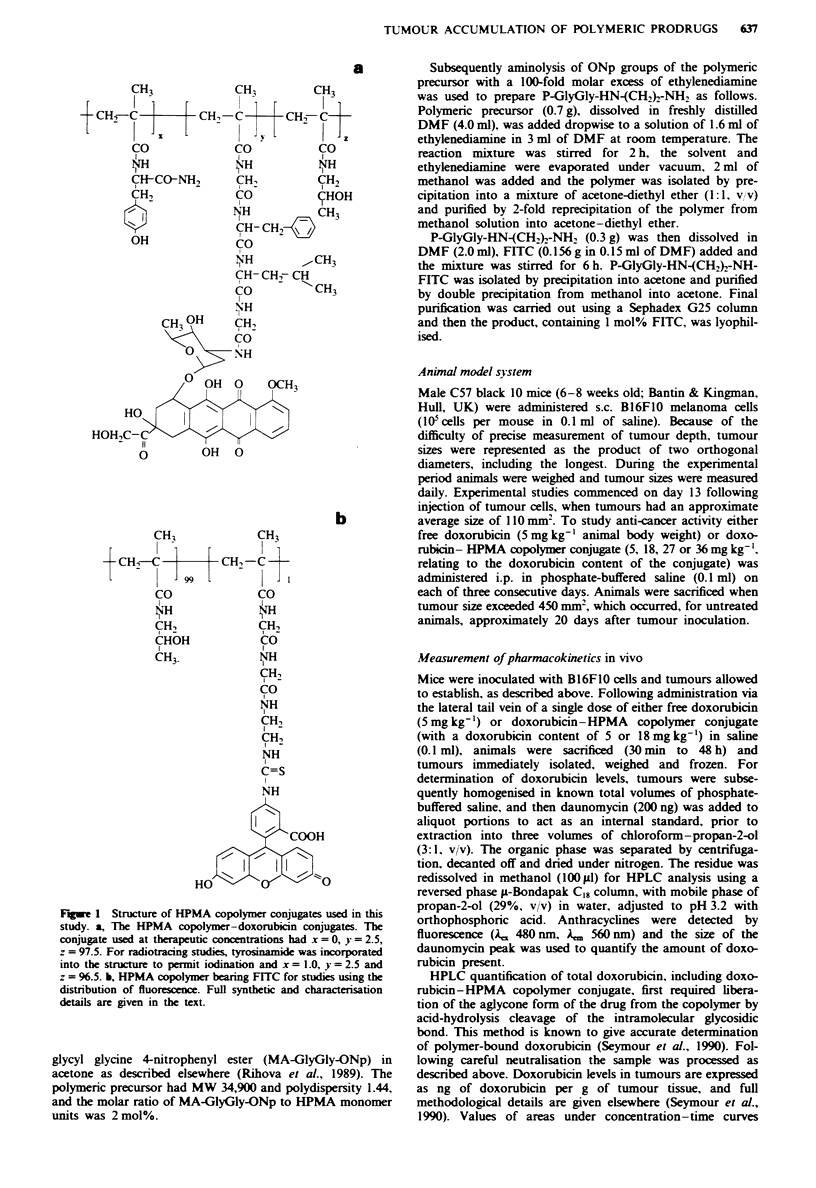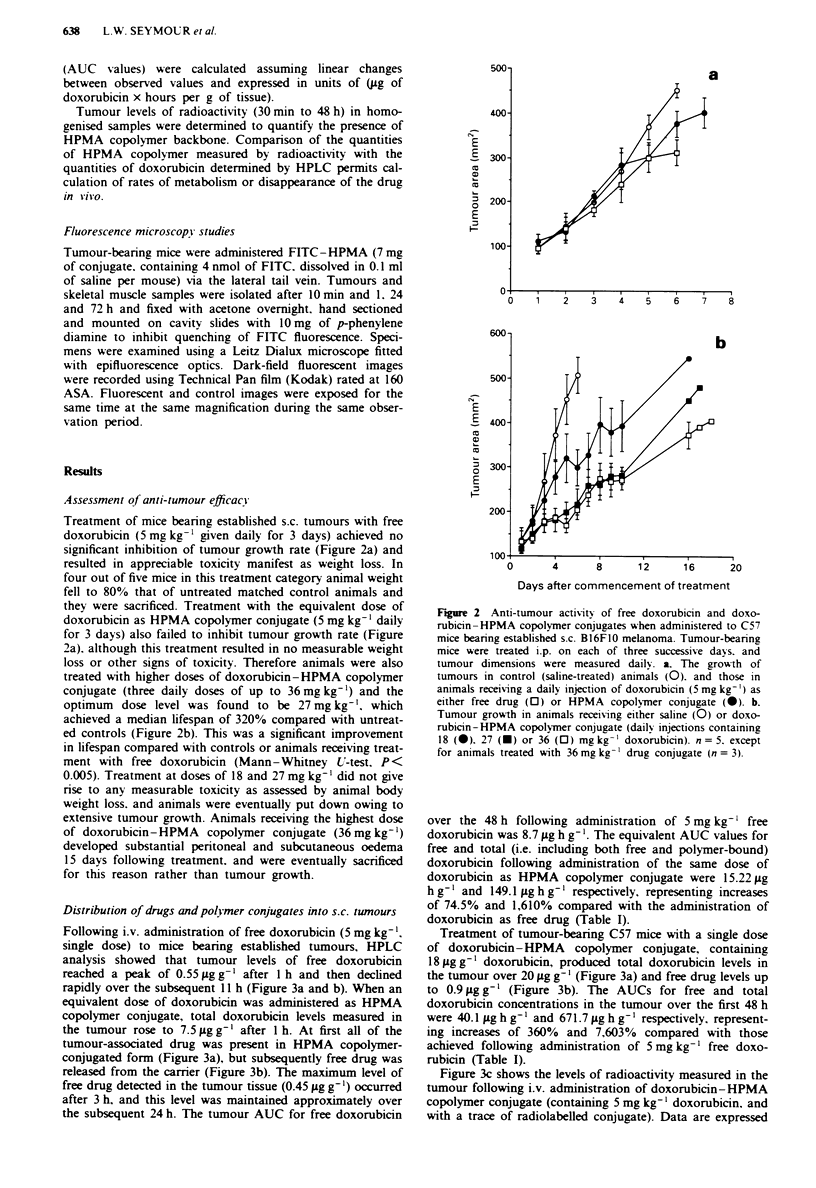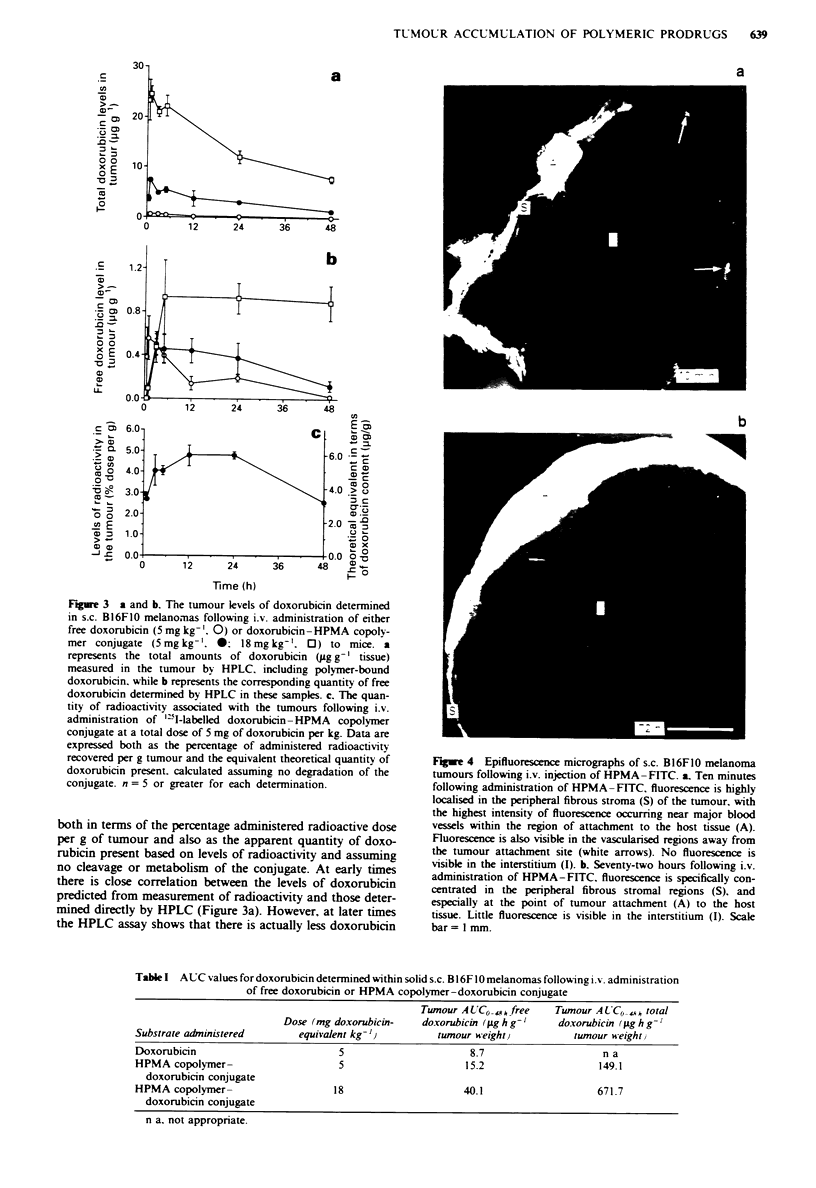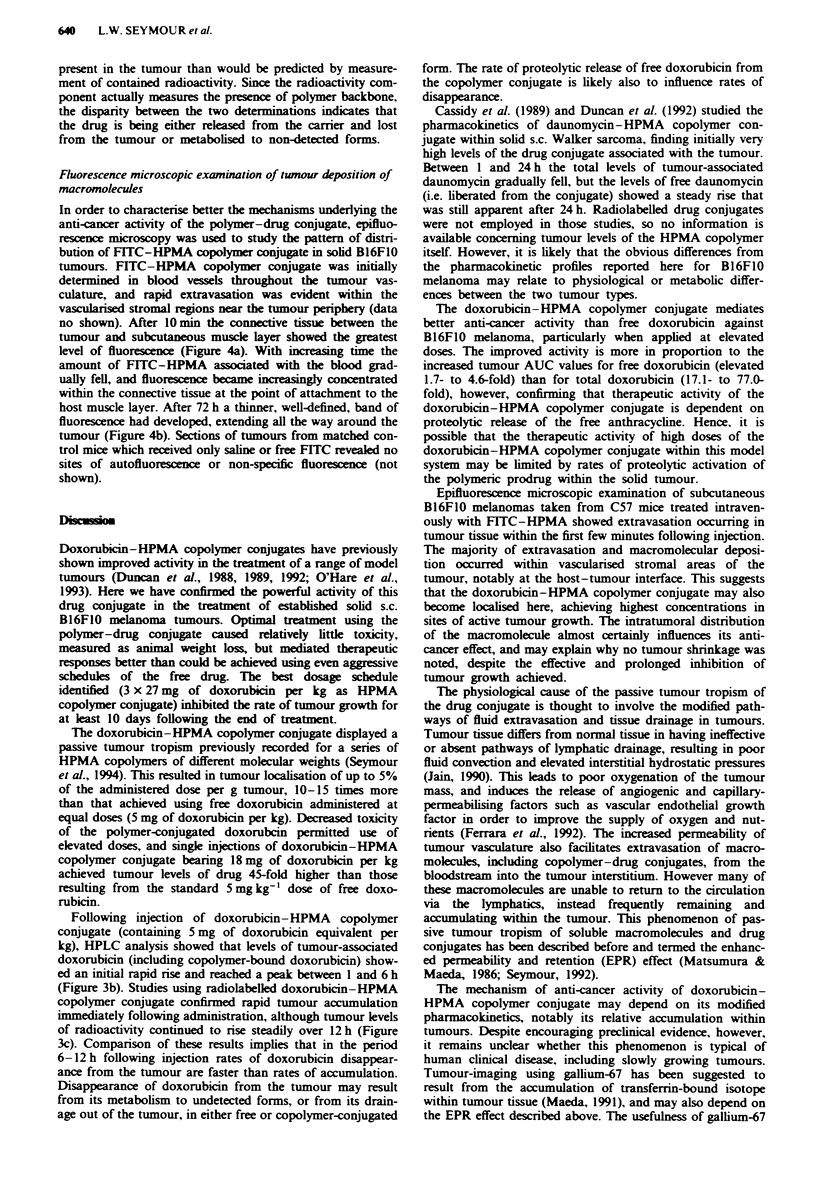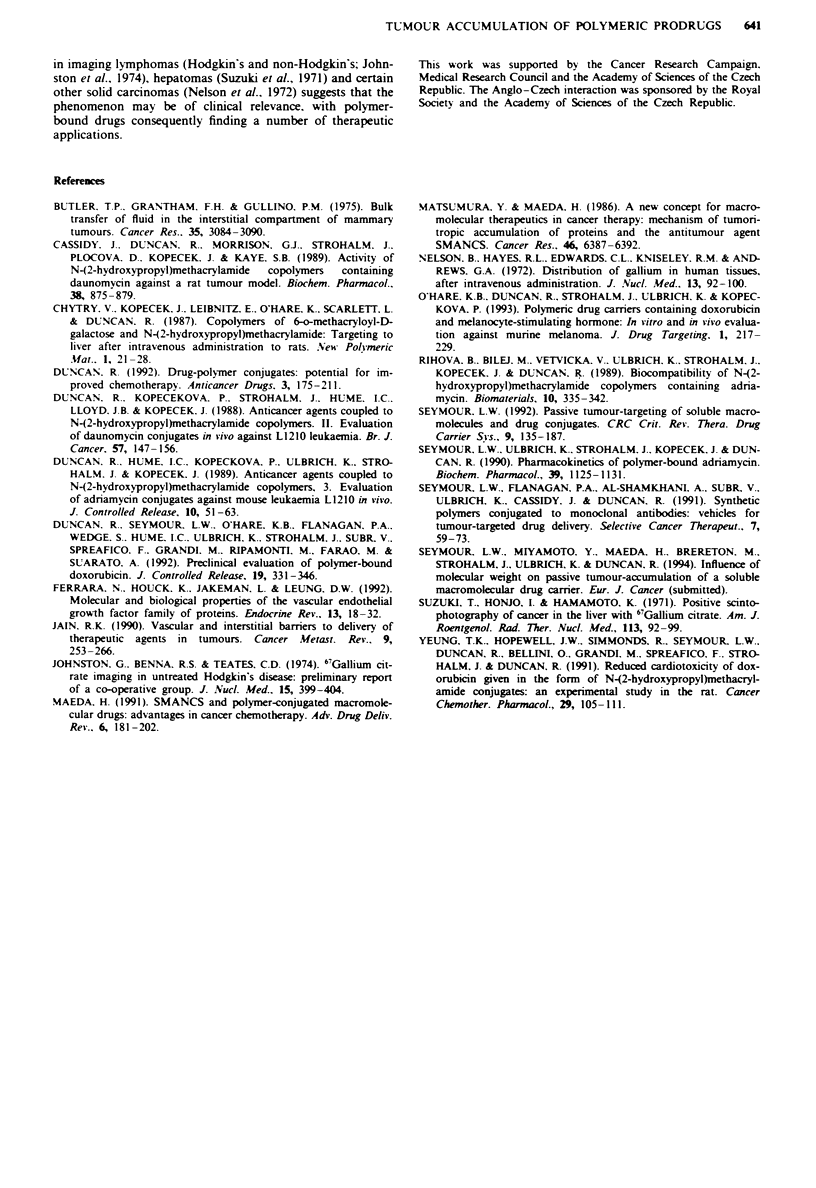# Tumour tropism and anti-cancer efficacy of polymer-based doxorubicin prodrugs in the treatment of subcutaneous murine B16F10 melanoma.

**DOI:** 10.1038/bjc.1994.363

**Published:** 1994-10

**Authors:** L. W. Seymour, K. Ulbrich, P. S. Steyger, M. Brereton, V. Subr, J. Strohalm, R. Duncan

**Affiliations:** Institute for Cancer Studies, University of Birmingham School of Medicine, UK.

## Abstract

**Images:**


Br. J. Cancer (1994), 70, 636-641 © Macmillan Press Ltd., 1994

### Tumour tropism and anti-cancer efficacy of polymer-based doxorubicin prodrugs in the treatment of subcutaneous murine B16F10 melanoma

L.W. Seymour[1], K. Ulbrich[2], P.S. Steyger[3], M. Brereton[1], V. Subr[2], J. Strohalm[2] & R. Duncan[4]

[1]*Institute for Cancer Studies, University of Birmingham School of Medicine, Birmingham B15 2TT, UK;* [2]*Institute of Macromolecular Chemistry, Academy of Sciences of the Czech Republic, Prague 6, The Czech Republic;* [3]*Department of Otolaryngology, University of Texas Health Science Centre, 7703 Floyd Curl Drive, San Antonio, Texas 78284-7777, USA;* [4]*Centre for Polymer Therapeutics, The School of Pharmacy, Brunswick Square, London WC1N 1AX, UK.*

**Summary** Doxorubicin (5 mg kg$^{-1}$) was administered intravenously to C57 mice bearing subcutaneous B16F10 melanomas, distributing into the tumour with an area under the concentration-time curve (0-48 h; AUC) of 8.7 μg h g$^{-1}$. Injection of doxorubicin-N-(2-hydroxypropyl)methacrylamide (HPMA) copolymer conjugate, containing 5 mg of doxorubicin equivalent per kg, mediated an AUC for free doxorubicin (i.e. doxorubicin released from the conjugate) of 15.2 μg h g$^{-1}$ and for total doxorubicin (i.e. free plus conjugated) of 149.1 μg h g$^{-1}$. An increased dose of doxorubicin-HPMA copolymer conjugate (18 mg of doxorubicin equivalent per kg) produced AUC values of 40.1 μg h g$^{-1}$ and 671.7 μg h g$^{-1}$ for free and total doxorubicin respectively. Hence administration of doxorubicin-HPMA copolymer conjugate achieved rises of 1.7- to 4.6-fold in tumour AUC (free doxorubicin) and 17.1- to 77.0-fold in tumour AUC (total doxorubicin). HPMA copolymers bearing fluorescein isothiocyanate accumulated in vascularised stromal regions, particularly in new growth sites at the tumour periphery. Treatment of mice with doxorubicin-HPMA copolymer conjugate achieved treated control lifespans up to 320% (three doses of 27 mg of doxorubicin equivalent per kg) compared with only 133% using aggressive regimens of free doxorubicin (3 × 5 mg kg$^{-1}$).

The pharmacokinetics of doxorubicin is modified following covalent conjugation to a copolymer based on N-(2-hydroxypropyl)methacrylamide (HPMA) (Figure 1) (Duncan, 1992). The doxorubicin-HPMA copolymer conjugate is unable to diffuse through cellular membranes and consequently displays a lower volume of distribution and longer plasma half-life than free doxorubicin (Seymour *et al.*, 1990). The poor membrane permeability of the conjugate also prevents its entry into cardiac tissue, reflected in decreased cardiotoxicity and permitting administration of increased doses of doxorubicin as a polymer conjugate (Yeung *et al.*, 1991).

The doxorubicin-HPMA copolymer conjugate has been shown to be essentially non-cytotoxic *in vitro* but to mediate anti-cancer activity against a range of animal tumour models *in vivo*, thought to result from proteolytic activation of the conjugate within tumour tissue (Duncan *et al.*, 1992). This proposed mode of action means that the activity of the drug conjugate is likely to be influenced by various biological factors, including biochemical and physiological features of the tumour.

Cassidy *et al.* (1989) and Duncan *et al.* (1992) used high-performance liquid chromatographic (HPLC) techniques to show that anthracycline-HPMA copolymer conjugates achieved high intratumoral levels of drug within established subcutaneous Walker sarcoma tumours, suggesting an ability of the drug conjugate to accumulate passively within solid tumours. However the Walker tumour is known to possess a relatively permeable vascular system (Butler *et al.*, 1975), and it is not clear whether passive tumour tropism and the consequent elevated tumour levels of drug is a significant component of the mechanism of action of these anthracycline-HPMA copolymer conjugates against other solid tumours.

In this study we have examined the activity of doxorubicin-HPMA copolymer conjugate against established s.c. tumours of murine melanoma B16F10. The pharmacokinetics and tumour accumulation of the conjugate have been studied using radiotracing and HPLC-based techniques, and the intratumoral distribution of fluorescein isothiocyanate-labelled HPMA copolymer (FITC-HPMA) has been examined by microscopy to identify sites of permeable vasculature and macromolecular deposition.

Correspondence: L. Seymour, Department of Clinical Oncology, Birmingham University School of Medicine, Edgbaston, Birmingham, B15 2TT, UK.
Received 7 March 1994; and in revised form 6 May 1994.

### Materials and methods

### *Reagents*

1-Amino-2-propanol, methacryloylchloride, dimethylformamide (DMF) and 4-nitrophenol were from Fluka, Buchs, Switzerland. Glycylphenylalanine and leucylglycine were from Cambridge Research Biochemicals, Northwich, UK. Doxorubicin was a kind gift from Farmitalia Carlo Erba, Milan, Italy, and FITC microscopy reagents and daunomycin were from Sigma, Poole, UK. HPLC solvents were from Fisons, Loughborough, UK.

### *Polymer conjugates*

Doxorubicin-HPMA copolymer conjugate (Figure 1a) was synthesised as described in full elsewhere (Rihova *et al.*, 1989); the material used in this study had a weight-average molecular weight (MW) of 24,000, and polydispersity (ratio of weight and number average-molecular weight) of 1.3, as determined by Sepharose 4B/6B gel permeation chromatography. Doxorubicin content was 7.2% (w/w) (2.5 mol%). To permit radioiodination an HPMA copolymer conjugate of doxorubicin was synthesised with similar specifications to contain also methacryloylated tyrosinamide as a comonomer (1.0 mol%, component *X*, Figure 1a) (Chytry *et al.*, 1987). Radiolabelling was performed with [$^{125}$I]iodide (Amersham International, UK) using a solid phase oxidising agent (Iodobeads, Pierce Chemicals, Rockford, USA) as described elsewhere (Seymour *et al.*, 1991). The radiolabelled conjugate had a specific radioactivity of 80 μCi mg$^{-1}$ conjugate and it was included at trace levels (1 μCi per mouse) in therapeutic doses of the unlabelled conjugate to allow monitoring of the fate of the polymer backbone.

FITC-HPMA was synthesised to permit monitoring of distribution of the polymer by fluorescence microscopy (Figure 1b). First a polymeric precursor P-GlyGly-ONp [P represents a poly(HPMA) backbone, GlyGly-ONp is the 4-nitrophenyl ester of glycyl glycine] was prepared by radical precipitation copolymerisation of HPMA with methacryloyl a b

**Figure 1** Structure of HPMA copolymer conjugates used in this study. **a**, The HPMA copolymer–doxorubicin conjugates. The conjugate used at therapeutic concentrations had $x = 0$, $y = 2.5$, $z = 97.5$. For radiotracing studies, tyrosinamide was incorporated into the structure to permit iodination and $x = 1.0$, $y = 2.5$ and $z = 96.5$. **b**, HPMA copolymer bearing FITC for studies using the distribution of fluorescence. Full synthetic and characterisation details are given in the text.

glycyl glycine 4-nitrophenyl ester (MA-GlyGly-ONp) in acetone as described elsewhere (Rihova *et al.*, 1989). The polymeric precursor had MW 34,900 and polydispersity 1.44, and the molar ratio of MA-GlyGly-ONp to HPMA monomer units was 2 mol%.

Subsequently aminolysis of ONp groups of the polymeric precursor with a 100-fold molar excess of ethylenediamine was used to prepare P-GlyGly-HN-$(CH_2)_2$-$NH_2$ as follows. Polymeric precursor (0.7 g), dissolved in freshly distilled DMF (4.0 ml), was added dropwise to a solution of 1.6 ml of ethylenediamine in 3 ml of DMF at room temperature. The reaction mixture was stirred for 2 h, the solvent and ethylenediamine were evaporated under vacuum, 2 ml of methanol was added and the polymer was isolated by precipitation into a mixture of acetone-diethyl ether (1:1, v/v) and purified by 2-fold reprecipitation of the polymer from methanol solution into acetone–diethyl ether.

P-GlyGly-HN-$(CH_2)_2$-$NH_2$ (0.3 g) was then dissolved in DMF (2.0 ml), FITC (0.156 g in 0.15 ml of DMF) added and the mixture was stirred for 6 h. P-GlyGly-HN-$(CH_2)_2$-NH-FITC was isolated by precipitation into acetone and purified by double precipitation from methanol into acetone. Final purification was carried out using a Sephadex G25 column and then the product, containing 1 mol% FITC, was lyophilised.

### *Animal model system*

Male C57 black 10 mice (6–8 weeks old; Bantin & Kingman, Hull, UK) were administered s.c. B16F10 melanoma cells ($10^5$ cells per mouse in 0.1 ml of saline). Because of the difficulty of precise measurement of tumour depth, tumour sizes were represented as the product of two orthogonal diameters, including the longest. During the experimental period animals were weighed and tumour sizes were measured daily. Experimental studies commenced on day 13 following injection of tumour cells, when tumours had an approximate average size of 110 $mm^2$. To study anti-cancer activity either free doxorubicin (5 mg $kg^{-1}$ animal body weight) or doxorubicin–HPMA copolymer conjugate (5, 18, 27 or 36 mg $kg^{-1}$, relating to the doxorubicin content of the conjugate) was administered i.p. in phosphate-buffered saline (0.1 ml) on each of three consecutive days. Animals were sacrificed when tumour size exceeded 450 $mm^2$, which occurred, for untreated animals, approximately 20 days after tumour inoculation.

### *Measurement of pharmacokinetics* in vivo

Mice were inoculated with B16F10 cells and tumours allowed to establish, as described above. Following administration via the lateral tail vein of a single dose of either free doxorubicin (5 mg $kg^{-1}$) or doxorubicin–HPMA copolymer conjugate (with a doxorubicin content of 5 or 18 mg $kg^{-1}$) in saline (0.1 ml), animals were sacrificed (30 min to 48 h) and tumours immediately isolated, weighed and frozen. For determination of doxorubicin levels, tumours were subsequently homogenised in known total volumes of phosphate-buffered saline, and then daunomycin (200 ng) was added to aliquot portions to act as an internal standard, prior to extraction into three volumes of chloroform–propan-2-ol (3:1, v/v). The organic phase was separated by centrifugation, decanted off and dried under nitrogen. The residue was redissolved in methanol (100 μl) for HPLC analysis using a reversed phase μ-Bondapak $C_{18}$ column, with mobile phase of propan-2-ol (29%, v/v) in water, adjusted to pH 3.2 with orthophosphoric acid. Anthracyclines were detected by fluorescence ($\lambda_{ex}$ 480 nm, $\lambda_{em}$ 560 nm) and the size of the daunomycin peak was used to quantify the amount of doxorubicin present.

HPLC quantification of total doxorubicin, including doxorubicin–HPMA copolymer conjugate, first required liberation of the aglycone form of the drug from the copolymer by acid-hydrolysis cleavage of the intramolecular glycosidic bond. This method is known to give accurate determination of polymer-bound doxorubicin (Seymour *et al.*, 1990). Following careful neutralisation the sample was processed as described above. Doxorubicin levels in tumours are expressed as ng of doxorubicin per g of tumour tissue, and full methodological details are given elsewhere (Seymour *et al.*, 1990). Values of areas under concentration–time curves

(AUC values) were calculated assuming linear changes between observed values and expressed in units of (µg of doxorubicin × hours per g of tissue).

Tumour levels of radioactivity (30 min to 48 h) in homogenised samples were determined to quantify the presence of HPMA copolymer backbone. Comparison of the quantities of HPMA copolymer measured by radioactivity with the quantities of doxorubicin determined by HPLC permits calculation of rates of metabolism or disappearance of the drug *in vivo*.

*Fluorescence microscopy studies*

Tumour-bearing mice were administered FITC-HPMA (7 mg of conjugate, containing 4 nmol of FITC, dissolved in 0.1 ml of saline per mouse) via the lateral tail vein. Tumours and skeletal muscle samples were isolated after 10 min and 1, 24 and 72 h and fixed with acetone overnight, hand sectioned and mounted on cavity slides with 10 mg of *p*-phenylene diamine to inhibit quenching of FITC fluorescence. Specimens were examined using a Leitz Dialux microscope fitted with epifluorescence optics. Dark-field fluorescent images were recorded using Technical Pan film (Kodak) rated at 160 ASA. Fluorescent and control images were exposed for the same time at the same magnification during the same observation period.

### Results

*Assessment of anti-tumour efficacy*

Treatment of mice bearing established s.c. tumours with free doxorubicin (5 mg kg$^{-1}$ given daily for 3 days) achieved no significant inhibition of tumour growth rate (Figure 2a) and resulted in appreciable toxicity manifest as weight loss. In four out of five mice in this treatment category animal weight fell to 80% that of untreated matched control animals and they were sacrificed. Treatment with the equivalent dose of doxorubicin as HPMA copolymer conjugate (5 mg kg$^{-1}$ daily for 3 days) also failed to inhibit tumour growth rate (Figure 2a), although this treatment resulted in no measurable weight loss or other signs of toxicity. Therefore animals were also treated with higher doses of doxorubicin-HPMA copolymer conjugate (three daily doses of up to 36 mg kg$^{-1}$) and the optimum dose level was found to be 27 mg kg$^{-1}$, which achieved a median lifespan of 320% compared with untreated controls (Figure 2b). This was a significant improvement in lifespan compared with controls or animals receiving treatment with free doxorubicin (Mann-Whitney *U*-test, $P<$ 0.005). Treatment at doses of 18 and 27 mg kg$^{-1}$ did not give rise to any measurable toxicity as assessed by animal body weight loss, and animals were eventually put down owing to extensive tumour growth. Animals receiving the highest dose of doxorubicin-HPMA copolymer conjugate (36 mg kg$^{-1}$) developed substantial peritoneal and subcutaneous oedema 15 days following treatment, and were eventually sacrificed for this reason rather than tumour growth.

*Distribution of drugs and polymer conjugates into s.c. tumours*

Following i.v. administration of free doxorubicin (5 mg kg$^{-1}$, single dose) to mice bearing established tumours, HPLC analysis showed that tumour levels of free doxorubicin reached a peak of 0.55 µg g$^{-1}$ after 1 h and then declined rapidly over the subsequent 11 h (Figure 3a and b). When an equivalent dose of doxorubicin was administered as HPMA copolymer conjugate, total doxorubicin levels measured in the tumour rose to 7.5 µg g$^{-1}$ after 1 h. At first all of the tumour-associated drug was present in HPMA copolymer-conjugated form (Figure 3a), but subsequently free drug was released from the carrier (Figure 3b). The maximum level of free drug detected in the tumour tissue (0.45 µg g$^{-1}$) occurred after 3 h, and this level was maintained approximately over the subsequent 24 h. The tumour AUC for free doxorubicin

**Figure 2** Anti-tumour activity of free doxorubicin and doxorubicin-HPMA copolymer conjugates when administered to C57 mice bearing established s.c. B16F10 melanoma. Tumour-bearing mice were treated i.p. on each of three successive days, and tumour dimensions were measured daily. **a**. The growth of tumours in control (saline-treated) animals (○), and those in animals receiving a daily injection of doxorubicin (5 mg kg$^{-1}$) as either free drug (□) or HPMA copolymer conjugate (●). **b**. Tumour growth in animals receiving either saline (○) or doxorubicin-HPMA copolymer conjugate (daily injections containing 18 (●), 27 (■) or 36 (□) mg kg$^{-1}$ doxorubicin). $n = 5$, except for animals treated with 36 mg kg$^{-1}$ drug conjugate ($n = 3$).

over the 48 h following administration of 5 mg kg$^{-1}$ free doxorubicin was 8.7 µg h g$^{-1}$. The equivalent AUC values for free and total (i.e. including both free and polymer-bound) doxorubicin following administration of the same dose of doxorubicin as HPMA copolymer conjugate were 15.22 µg h g$^{-1}$ and 149.1 µg h g$^{-1}$ respectively, representing increases of 74.5% and 1,610% compared with the administration of doxorubicin as free drug (Table I).

Treatment of tumour-bearing C57 mice with a single dose of doxorubicin-HPMA copolymer conjugate, containing 18 µg g$^{-1}$ doxorubicin, produced total doxorubicin levels in the tumour over 20 µg g$^{-1}$ (Figure 3a) and free drug levels up to 0.9 µg g$^{-1}$ (Figure 3b). The AUCs for free and total doxorubicin concentrations in the tumour over the first 48 h were 40.1 µg h g$^{-1}$ and 671.7 µg h g$^{-1}$ respectively, representing increases of 360% and 7,603% compared with those achieved following administration of 5 mg kg$^{-1}$ free doxorubicin (Table I).

Figure 3c shows the levels of radioactivity measured in the tumour following i.v. administration of doxorubicin-HPMA copolymer conjugate (containing 5 mg kg$^{-1}$ doxorubicin, and with a trace of radiolabelled conjugate). Data are expressed

**Figure 3** **a** and **b**. The tumour levels of doxorubicin determined in s.c. B16F10 melanomas following i.v. administration of either free doxorubicin (5 mg $kg^{-1}$, ○) or doxorubicin-HPMA copolymer conjugate (5 mg $kg^{-1}$, ●; 18 mg $kg^{-1}$, □) to mice. **a** represents the total amounts of doxorubicin (µg $g^{-1}$ tissue) measured in the tumour by HPLC, including polymer-bound doxorubicin, while **b** represents the corresponding quantity of free doxorubicin determined by HPLC in these samples. **c**, The quantity of radioactivity associated with the tumours following i.v. administration of $^{125}$I-labelled doxorubicin-HPMA copolymer conjugate at a total dose of 5 mg of doxorubicin per kg. Data are expressed both as the percentage of administered radioactivity recovered per g tumour and the equivalent theoretical quantity of doxorubicin present, calculated assuming no degradation of the conjugate. $n = 5$ or greater for each determination.

both in terms of the percentage administered radioactive dose per g of tumour and also as the apparent quantity of doxorubicin present based on levels of radioactivity and assuming no cleavage or metabolism of the conjugate. At early times there is close correlation between the levels of doxorubicin predicted from measurement of radioactivity and those determined directly by HPLC (Figure 3a). However, at later times the HPLC assay shows that there is actually less doxorubicin

**Figure 4** Epifluorescence micrographs of s.c. B16F10 melanoma tumours following i.v. injection of HPMA-FITC. **a**, Ten minutes following administration of HPMA-FITC, fluorescence is highly localised in the peripheral fibrous stroma (S) of the tumour, with the highest intensity of fluorescence occurring near major blood vessels within the region of attachment to the host tissue (A). Fluorescence is also visible in the vascularised regions away from the tumour attachment site (white arrows). No fluorescence is visible in the interstitium (I). **b**, Seventy-two hours following i.v. administration of HPMA-FITC, fluorescence is specifically concentrated in the peripheral fibrous stromal regions (S), and especially at the point of tumour attachment (A) to the host tissue. Little fluorescence is visible in the interstitium (I). Scale bar = 1 mm.

**Table I** AUC values for doxorubicin determined within solid s.c. B16F10 melanomas following i.v. administration of free doxorubicin or HPMA copolymer-doxorubicin conjugate

| *Substrate administered* | *Dose (mg doxorubicin-equivalent $kg^{-1}$)* | *Tumour $AUC_{0-48h}$ free doxorubicin (µg h $g^{-1}$ tumour weight)* | *Tumour $AUC_{0-48h}$ total doxorubicin (µg h $g^{-1}$ tumour weight)* |
|---|---|---|---|
| Doxorubicin | 5 | 8.7 | n a |
| HPMA copolymer-doxorubicin conjugate | 5 | 15.2 | 149.1 |
| HPMA copolymer-doxorubicin conjugate | 18 | 40.1 | 671.7 |

n a, not appropriate.

present in the tumour than would be predicted by measurement of contained radioactivity. Since the radioactivity component actually measures the presence of polymer backbone, the disparity between the two determinations indicates that the drug is being either released from the carrier and lost from the tumour or metabolised to non-detected forms.

*Fluorescence microscopic examination of tumour deposition of macromolecules*

In order to characterise better the mechanisms underlying the anti-cancer activity of the polymer-drug conjugate, epifluorescence microscopy was used to study the pattern of distribution of FITC-HPMA copolymer conjugate in solid B16F10 tumours. FITC-HPMA copolymer conjugate was initially determined in blood vessels throughout the tumour vasculature, and rapid extravasation was evident within the vascularised stromal regions near the tumour periphery (data no shown). After 10 min the connective tissue between the tumour and subcutaneous muscle layer showed the greatest level of fluorescence (Figure 4a). With increasing time the amount of FITC-HPMA associated with the blood gradually fell, and fluorescence became increasingly concentrated within the connective tissue at the point of attachment to the host muscle layer. After 72 h a thinner, well-defined, band of fluorescence had developed, extending all the way around the tumour (Figure 4b). Sections of tumours from matched control mice which received only saline or free FITC revealed no sites of autofluorescence or non-specific fluorescence (not shown).

### Discussion

Doxorubicin-HPMA copolymer conjugates have previously shown improved activity in the treatment of a range of model tumours (Duncan *et al.*, 1988, 1989, 1992; O'Hare *et al.*, 1993). Here we have confirmed the powerful activity of this drug conjugate in the treatment of established solid s.c. B16F10 melanoma tumours. Optimal treatment using the polymer-drug conjugate caused relatively little toxicity, measured as animal weight loss, but mediated therapeutic responses better than could be achieved using even aggressive schedules of the free drug. The best dosage schedule identified (3 × 27 mg of doxorubicin per kg as HPMA copolymer conjugate) inhibited the rate of tumour growth for at least 10 days following the end of treatment.

The doxorubicin-HPMA copolymer conjugate displayed a passive tumour tropism previously recorded for a series of HPMA copolymers of different molecular weights (Seymour *et al.*, 1994). This resulted in tumour localisation of up to 5% of the administered dose per g tumour, 10-15 times more than that achieved using free doxorubicin administered at equal doses (5 mg of doxorubicin per kg). Decreased toxicity of the polymer-conjugated doxorubcin permitted use of elevated doses, and single injections of doxorubicin-HPMA copolymer conjugate bearing 18 mg of doxorubicin per kg achieved tumour levels of drug 45-fold higher than those resulting from the standard 5 mg kg$^{-1}$ dose of free doxorubicin.

Following injection of doxorubicin-HPMA copolymer conjugate (containing 5 mg of doxorubicin equivalent per kg), HPLC analysis showed that levels of tumour-associated doxorubicin (including copolymer-bound doxorubicin) showed an initial rapid rise and reached a peak between 1 and 6 h (Figure 3b). Studies using radiolabelled doxorubicin-HPMA copolymer conjugate confirmed rapid tumour accumulation immediately following administration, although tumour levels of radioactivity continued to rise steadily over 12 h (Figure 3c). Comparison of these results implies that in the period 6-12 h following injection rates of doxorubicin disappearance from the tumour are faster than rates of accumulation. Disappearance of doxorubicin from the tumour may result from its metabolism to undetected forms, or from its drainage out of the tumour, in either free or copolymer-conjugated form. The rate of proteolytic release of free doxorubicin from the copolymer conjugate is likely also to influence rates of disappearance.

Cassidy *et al.* (1989) and Duncan *et al.* (1992) studied the pharmacokinetics of daunomycin-HPMA copolymer conjugate within solid s.c. Walker sarcoma, finding initially very high levels of the drug conjugate associated with the tumour. Between 1 and 24 h the total levels of tumour-associated daunomycin gradually fell, but the levels of free daunomycin (i.e. liberated from the conjugate) showed a steady rise that was still apparent after 24 h. Radiolabelled drug conjugates were not employed in those studies, so no information is available concerning tumour levels of the HPMA copolymer itself. However, it is likely that the obvious differences from the pharmacokinetic profiles reported here for B16F10 melanoma may relate to physiological or metabolic differences between the two tumour types.

The doxorubicin-HPMA copolymer conjugate mediates better anti-cancer activity than free doxorubicin against B16F10 melanoma, particularly when applied at elevated doses. The improved activity is more in proportion to the increased tumour AUC values for free doxorubicin (elevated 1.7- to 4.6-fold) than for total doxorubicin (17.1- to 77.0-fold), however, confirming that therapeutic activity of the doxorubicin-HPMA copolymer conjugate is dependent on proteolytic release of the free anthracycline. Hence, it is possible that the therapeutic activity of high doses of the doxorubicin-HPMA copolymer conjugate within this model system may be limited by rates of proteolytic activation of the polymeric prodrug within the solid tumour.

Epifluorescence microscopic examination of subcutaneous B16F10 melanomas taken from C57 mice treated intravenously with FITC-HPMA showed extravasation occurring in tumour tissue within the first few minutes following injection. The majority of extravasation and macromolecular deposition occurred within vascularised stromal areas of the tumour, notably at the host-tumour interface. This suggests that the doxorubicin-HPMA copolymer conjugate may also become localised here, achieving highest concentrations in sites of active tumour growth. The intratumoral distribution of the macromolecule almost certainly influences its anti-cancer effect, and may explain why no tumour shrinkage was noted, despite the effective and prolonged inhibition of tumour growth achieved.

The physiological cause of the passive tumour tropism of the drug conjugate is thought to involve the modified pathways of fluid extravasation and tissue drainage in tumours. Tumour tissue differs from normal tissue in having ineffective or absent pathways of lymphatic drainage, resulting in poor fluid convection and elevated interstitial hydrostatic pressures (Jain, 1990). This leads to poor oxygenation of the tumour mass, and induces the release of angiogenic and capillary-permeabilising factors such as vascular endothelial growth factor in order to improve the supply of oxygen and nutrients (Ferrara *et al.*, 1992). The increased permeability of tumour vasculature also facilitates extravasation of macromolecules, including copolymer-drug conjugates, from the bloodstream into the tumour interstitium. However many of these macromolecules are unable to return to the circulation via the lymphatics, instead frequently remaining and accumulating within the tumour. This phenomenon of passive tumour tropism of soluble macromolecules and drug conjugates has been described before and termed the enhanced permeability and retention (EPR) effect (Matsumura & Maeda, 1986; Seymour, 1992).

The mechanism of anti-cancer activity of doxorubicin-HPMA copolymer conjugate may depend on its modified pharmacokinetics, notably its relative accumulation within tumours. Despite encouraging preclinical evidence, however, it remains unclear whether this phenomenon is typical of human clinical disease, including slowly growing tumours. Tumour-imaging using gallium-67 has been suggested to result from the accumulation of transferrin-bound isotope within tumour tissue (Maeda, 1991), and may also depend on the EPR effect described above. The usefulness of gallium-67

in imaging lymphomas (Hodgkin's and non-Hodgkin's; Johnston *et al.*, 1974), hepatomas (Suzuki *et al.*, 1971) and certain other solid carcinomas (Nelson *et al.*, 1972) suggests that the phenomenon may be of clinical relevance, with polymer-bound drugs consequently finding a number of therapeutic applications.

This work was supported by the Cancer Research Campaign, Medical Research Council and the Academy of Sciences of the Czech Republic. The Anglo-Czech interaction was sponsored by the Royal Society and the Academy of Sciences of the Czech Republic.

**References**

BUTLER, T.P., GRANTHAM, F.H. & GULLINO, P.M. (1975). Bulk transfer of fluid in the interstitial compartment of mammary tumours. *Cancer Res.*, **35**, 3084-3090.

CASSIDY, J., DUNCAN, R., MORRISON, G.J., STROHALM, J., PLOCOVA, D., KOPECEK, J. & KAYE, S.B. (1989). Activity of N-(2-hydroxypropyl)methacrylamide copolymers containing daunomycin against a rat tumour model. *Biochem. Pharmacol.*, **38**, 875-879.

CHYTRY, V., KOPECEK, J., LEIBNITZ, E., O'HARE, K., SCARLETT, L. & DUNCAN, R. (1987). Copolymers of 6-o-methacryloyl-D-galactose and N-(2-hydroxypropyl)methacrylamide: Targeting to liver after intravenous administration to rats. *New Polymeric Mat.*, **1**, 21-28.

DUNCAN, R. (1992). Drug-polymer conjugates: potential for improved chemotherapy. *Anticancer Drugs*, **3**, 175-211.

DUNCAN, R., KOPECEKOVA, P., STROHALM, J., HUME, I.C., LLOYD, J.B. & KOPECEK, J. (1988). Anticancer agents coupled to N-(2-hydroxypropyl)methacrylamide copolymers. II. Evaluation of daunomycin conjugates *in vivo* against L1210 leukaemia. *Br. J. Cancer*, **57**, 147-156.

DUNCAN, R., HUME, I.C., KOPECKOVA, P., ULBRICH, K., STROHALM, J. & KOPECEK, J. (1989). Anticancer agents coupled to N-(2-hydroxypropyl)methacrylamide copolymers, 3. Evaluation of adriamycin conjugates against mouse leukaemia L1210 *in vivo*. *J. Controlled Release*, **10**, 51-63.

DUNCAN, R., SEYMOUR, L.W., O'HARE, K.B., FLANAGAN, P.A., WEDGE, S., HUME, I.C., ULBRICH, K., STROHALM, J., SUBR, V., SPREAFICO, F., GRANDI, M., RIPAMONTI, M., FARAO, M. & SUARATO, A. (1992). Preclinical evaluation of polymer-bound doxorubicin. *J. Controlled Release*, **19**, 331-346.

FERRARA, N., HOUCK, K., JAKEMAN, L. & LEUNG, D.W. (1992). Molecular and biological properties of the vascular endothelial growth factor family of proteins. *Endocrine Rev.*, **13**, 18-32.

JAIN, R.K. (1990). Vascular and interstitial barriers to delivery of therapeutic agents in tumours. *Cancer Metast. Rev.*, **9**, 253-266.

JOHNSTON, G., BENNA, R.S. & TEATES, C.D. (1974). $^{67}$Gallium citrate imaging in untreated Hodgkin's disease: preliminary report of a co-operative group. *J. Nucl. Med.*, **15**, 399-404.

MAEDA, H. (1991). SMANCS and polymer-conjugated macromolecular drugs: advantages in cancer chemotherapy. *Adv. Drug Deliv. Rev.*, **6**, 181-202.

MATSUMURA, Y. & MAEDA, H. (1986). A new concept for macromolecular therapeutics in cancer therapy: mechanism of tumoritropic accumulation of proteins and the antitumour agent SMANCS. *Cancer Res.*, **46**, 6387-6392.

NELSON, B., HAYES, R.L., EDWARDS, C.L., KNISELEY, R.M. & ANDREWS, G.A. (1972). Distribution of gallium in human tissues, after intravenous administration. *J. Nucl. Med.*, **13**, 92-100.

O'HARE, K.B., DUNCAN, R., STROHALM, J., ULBRICH, K. & KOPECKOVA, P. (1993). Polymeric drug carriers containing doxorubicin and melanocyte-stimulating hormone: *In vitro* and *in vivo* evaluation against murine melanoma. *J. Drug Targeting*, **1**, 217-229.

RIHOVA, B., BILEJ, M., VETVICKA, V., ULBRICH, K., STROHALM, J., KOPECEK, J. & DUNCAN, R. (1989). Biocompatibility of N-(2-hydroxypropyl)methacrylamide copolymers containing adriamycin. *Biomaterials*, **10**, 335-342.

SEYMOUR, L.W. (1992). Passive tumour-targeting of soluble macromolecules and drug conjugates. *CRC Crit. Rev. Thera. Drug Carrier Sys.*, **9**, 135-187.

SEYMOUR, L.W., ULBRICH, K., STROHALM, J., KOPECEK, J. & DUNCAN, R. (1990). Pharmacokinetics of polymer-bound adriamycin. *Biochem. Pharmacol.*, **39**, 1125-1131.

SEYMOUR, L.W., FLANAGAN, P.A., AL-SHAMKHANI, A., SUBR, V., ULBRICH, K., CASSIDY, J. & DUNCAN, R. (1991). Synthetic polymers conjugated to monoclonal antibodies: vehicles for tumour-targeted drug delivery. *Selective Cancer Therapeut.*, **7**, 59-73.

SEYMOUR, L.W., MIYAMOTO, Y., MAEDA, H., BRERETON, M., STROHALM, J., ULBRICH, K. & DUNCAN, R. (1994). Influence of molecular weight on passive tumour-accumulation of a soluble macromolecular drug carrier. *Eur. J. Cancer* (submitted).

SUZUKI, T., HONJO, I. & HAMAMOTO, K. (1971). Positive scintophotography of cancer in the liver with $^{67}$Gallium citrate. *Am. J. Roentgenol. Rad. Ther. Nucl. Med.*, **113**, 92-99.

YEUNG, T.K., HOPEWELL, J.W., SIMMONDS, R., SEYMOUR, L.W., DUNCAN, R., BELLINI, O., GRANDI, M., SPREAFICO, F., STROHALM, J. & DUNCAN, R. (1991). Reduced cardiotoxicity of doxorubicin given in the form of N-(2-hydroxypropyl)methacrylamide conjugates: an experimental study in the rat. *Cancer Chemother. Pharmacol.*, **29**, 105-111.